# Self-referential and social saliency information influences memory following attention orienting

**DOI:** 10.3389/fpsyg.2023.1092512

**Published:** 2023-03-22

**Authors:** Shuo Zhao, Shota Uono, Rong Qing Hu, Sayaka Yoshimura, Motomi Toichi

**Affiliations:** ^1^School of Psychology, Shenzhen University, Shenzhen, Guangdong, China; ^2^Department of Human Health Sciences, Faculty of Medicine, Kyoto University, Kyoto, Japan; ^3^Department of Developmental Disorders, National Institute of Mental Health, National Center of Neurology and Psychiatry, Tokyo, Japan; ^4^Shenzhen Polytechnic, Shenzhen, Guangdong, China

**Keywords:** self-referential processing, social salience, attention orienting, memory, emotion

## Abstract

Self-referential information is a processing priority in individuals. Whether or how self-referential information plays a role in attention orienting by modulating memory encoding during attention orienting is presently unknown. First, we investigated this role with self-referential processing for words. Participants were trained to associate two cues (red and green arrows) with social labels (the words “self” and “other” in Experiment 1). Then, participants performed a cueing task to determine whether various targets were presented at a right or left location. Finally, a recognition task of target items was implemented to examine the influence of arrow cues on memory. Second, given that the difference in social salience also exists between self-and other-referential processing, we investigate whether the same effect as the self-referential processing of words exists for emotional faces with high social salience and regardless of emotional valence (a high and a low social salience in Experiment 2A; and a positive and a negative emotional face in Experiment 2B). The results showed that self-referential and emotional cues, irrespective of their emotional valence, enhance memory for the indicated target objects across experiments. This suggests that automatic prioritization of social salience for self-referential words or emotional faces plays an important role in subsequent cognitive processing through attention orienting to influence memory.

## Introduction

During the past three decades, many studies have shown that the concept of the self is unique to the individual and is inherently a social construct that serves as a stable anchor for understanding other people (Sui and Gu, 2017). Self-referential information is processed with priority, evinced in the greater recall rate and faster response speed to information (e.g., Rogers et al., 1977). Furthermore, the importance of self-relevant components has been highlighted during perception, such as responding a “self” vs. “other” word (e.g., Sui et al., 2009; Williams et al., 2018), and cognitive processing, such as working memory and decision making (e.g., Yin et al., 2019; see Sui and Humphreys (2015) for a review). Despite a large body of evidence, it remains unknown whether self-referential processing works in a qualitatively distinguishable manner from other-referential processing during attention orienting.

Attention orienting allows us to preferentially process and learn information from our own point of reference (Bayliss and Tipper, 2006) and to understand the other person’s inner state by following the orientation of another individual’s eye gaze, therefore influencing memory (e.g., Dodd et al., 2012) and affective judgements for the indicated objects (e.g., Bayliss and Tipper, 2006). Although response times (RTs) in attention orienting could also be influenced by arrows as cues, eye gaze, unlike arrows as cues reflects a qualitatively human ability to modulate the depth of encoding for the targets underlying social communication. Specifically, attention orienting by gaze but not arrow cues has been found to enhance memory for the indicated (valid) items even when participants are not explicitly attempting to memorize the items (Dodd et al., 2012). Using a cueing paradigm (Dodd et al., 2012), participants were asked to focus on a word. Then, a memory task was administered. Although the participants were not instructed to memorize the word, an enhanced memory for the indicated word was found by gaze but not arrow cues. This might reflect an incidental episodic memory for the indicated targets facilitated by gaze but not arrow cues in attention orienting. Therefore, to determine whether a qualitatively distinguishable manner is influenced by self-referential processing, it is important to investigate whether the self works to aid the depth of encoding for the targets (i.e., memory) indicated by the cues during attention orienting.

Self-referential processing has been investigated in attention orienting under a cueing paradigm (e.g., Sui et al., 2009; Zhao et al., 2015). Sui et al. (2009) developed a method to modulate the self-referentiality of cues. First, in the training task, participants learn associations between a specific arrow shape and themselves, treating it as a self-referential cue, and between a different arrow shape and a friend, serving as an other-referential cue. Under a subsequent cueing task, the study showed that self-referential arrow cues induce a faster response than other-referential arrow cues when the cue direction and the target location were incongruent at a short cue-target stimulus onset asynchrony (SOA), which indicates that attention more rapidly disengages from the cued spatial location to respond to a target in the self-referential condition.

Zhao et al. (2015) also used the same method to associate cues with the self and others. Participants were first trained to associate two cues (a red and green arrow in Experiment 1A and two different faces in Experiment 1B) with distinct words (“self” and “other”) and then used two cues (self-referential and other-referential) and two types of sound (voice and tone as target) in the cueing task. The results found that a large cueing effect could be elicited by self-referential but not other-referential cues on a specific target stimulus (i.e., a voice but not a tone). These findings reflected that attention orienting could be modulated by self-referential processing for words. Interestingly, Zhao et al. (2015), in Experiment 2, also found that self-referential arrow cues and other-referential gaze cues showed the same pattern of attention orienting with commonly used gaze and arrow cues, respectively (cf. Zhao et al., 2014). Self-referential cues could modulate automatic attention orienting by centrally presented cues. However, it remains unknown whether self-reference works in a qualitatively distinguishable manner to influence and modulate accompanying cognitive processing with attention orienting, such as memory. Thus, this study first tested whether self-referential processing for words aids memory for the items indicated by the cues during attention orienting in Experiment 1. Given that individuals are commonly highly familiar with self-referential stimuli such as their face or name, it is possible that participant memory was enhanced by familiarity rather than the self-referential effect in attention orienting. Based on a previous experimental design to avoid causing a familiarity effect and examine how attention orienting was influenced by self-referential processing (Zhao et al., 2015), participants in a training task were first asked to establish an association between two cues (red and green arrows) and “self” and “other” words (i.e., encoding the relevant arrow stimuli). Previous studies (e.g., Sui et al., 2009; Williams et al., 2018) have shown that self-referential effects exist in “self” versus “other” words. If a low error rate in self-pairs and other pairs were found, the association between arrow cues and “self” and “other” words would be firmly established in the training task. Subsequently, a cueing task using the self-and other-referential arrows required participants to determine whether various targets (a set of meaningless shapes) were presented at a right or left location. Then, a recognition task was implemented to examine the influence of self-and other-referential arrow cues on memory for target shapes during attention orienting. We hypothesized that the number of memorized items indicated by the cues could be enhanced when participants perceived arrow cues associated with “self” words (a high self-referential degree) but not cues associated with “other” words (a low self-referential degree) in Experiment 1.

Second, there is a possibility that a difference between self-and other-referential processing is also reflected in the level of social salience (Sui et al., 2012; Scheller and Sui, 2022). Social salience is inherent in self-referential as well as emotional information such as what is attractive or dangerous. Some studies have shown that self-referential (e.g., Sui et al., 2009) as well as emotional information modulate attention orienting (e.g., Lassalle and Itier, 2015) and memory performance (e.g., Tyng et al., 2017). Other studies suggest the differential role of self-referential and emotional processing (Stolte et al., 2017). To determine whether the social salience of stimuli can influence accompanying cognitive processing with attention orienting, we addressed whether another person’s emotional faces with high social salience, which is defined by the valence and emotional arousal of an experience (Alger and Payne, 2016), had the same influence on cognitive processing during attention orienting as the self-relevant words. The level of social salience for perceiving another person’s emotional face determines how we perceive and attend to the world, and how we behave. For example, happy faces are friendly related stimuli, and fearful faces are threat stimuli, both of which may present higher levels of social salience and be more subjected to attentional biases [A review for Yiend (2010)]. Thus, in Experiment 2A, two cues (red and green arrows) were associated with faces with a happy and a neutral face with a straight gaze in the training task. If a low error rate was found for both positive and neutral face pairs, the association between arrow cues and positive and neutral faces was firmly established in the training task. Then, the cueing task and the recognition task were implemented. However, this experiment cannot exclude the possibility that the difference in memory performance under the recognition task might be explained by only positive emotional valence of the word “self” and happy faces.

In Experiment 2B, we manipulated two cues (red and green arrows) so that they were associated with a positive and a negative emotional face (i.e., a happy and a fearful face) in the training task. If a low error rate were found for both positive and negative emotional face pairs, the association between arrow cues and positive and negative emotional faces would be firmly established in the training task. Moreover, given that both emotional faces have a high magnitude of social salience, we presumed a large difference in emotional valence but a similar degree of social salience between happy and fearful faces with a straight gaze. Thus, if the magnitude of social salience but not positive emotional valence manifests a qualitatively different function to modulate participants’ cognitive processing during attention orienting, we hypothesized that the number of memorized items indicated by the cues could be enhanced when participants perceived arrow cues associated with happy faces (a high magnitude of social salience and a positive emotional valence) but not cues associated with neutral faces (a low magnitude of social salience degree and a neutral emotional valance) in Experiment 2A and no difference between when participants perceived cues associated with happy (a high magnitude of social salience degree and a positive emotional valance) and fearful faces (a high magnitude of social salience and a negative emotional valance) in Experiment 2B.

## Experiment 1

The purpose of Experiment 1 was to determine whether self-referential processing of words can aid in influencing memory during attention orienting. In the training task, participants were first trained to associate arrow cues and two different words representing “self” or “other” words. To this end, a cueing paradigm was implemented, in which using the arrows associated with “self” and “other” words as cues required participants to determine whether various targets were presented at a right or left location. Finally, the recognition task was used to examine the effect of self-referential processing on memory, even when participants did not attempt to memorize items (i.e., target shapes under the cueing task). We hypothesized that the number of memorized items indicated by the cues could be enhanced when participants perceived arrow cues associated with “self” words but not cues associated with “other” words.

### Materials and methods

#### Participants

Fifty-four Japanese students (mean age ± SD, 21.3 ± 0.47 years; 25 males) participated in Experiment 1. The effect size in a comparable previous study (Experiment 3 in Dodd et al., 2012) was 0.1 (62 participants). With a desired power (0.95) for detecting the effect size (*f* = 0.21) at an alpha level of 0.05, we needed a minimum sample of 52 participants, which was calculated by G*Power (Faul et al., 2007). The dominant hand of the participants was evaluated by the Edinburgh Handedness Inventory (Oldfield, 1971). All participants were right-handed. There was no overlap in participants across experiments, and all participants reported normal colour vision and normal or corrected-to-normal visual acuity. This study was approved by the local ethics committee, and all procedures complied with the ethical standards of the 1964 Declaration of Helsinki regarding the treatment of human participants in research. This study was not preregistered.

#### Stimuli

To compare our results with those of a previous study (Zhao et al., 2015), we used the same stimuli as those in the training task. In the training task, the stimuli used were illustrated. A red or green arrow (8.3° wide × 3.0° high) was displayed above the fixation cross, and a “self” (自分) or “other” (他人) (6.8° wide × 3.0° high) word was presented below the fixation cross. The red and green arrows implemented in the cueing task were the same as those implemented in the training task.

In the cueing and recognition task, the target stimuli were drawn from the set of novel meaningless closed shape contours (3.8° ~ 3.9° wide × 3.2° ~ 5.8° high) developed by Endo et al. (2003), which were difficult to verbalize. All stimulus contours were drawn with black outlines and a white background. According to the perceptual preference score (Endo et al., 2003), we divided 64 shape stimuli into four sets, for which there was no difference in perceptual preference (Supplementary Result S1; Supplementary Table S1). We used two sets (one set with the self-referential arrow condition and the other set with the other-referential arrow condition) in the cueing task and the other two sets (one was paired with the self-referential set and the other one was paired with the other-referential set as a novel item) in the recognition task, and these assignments were counterbalanced across participants in the cueing task. The centre of the shape stimuli appeared 9.6° to the left or the right of the cue.

#### Apparatus

The stimuli and fixation icons were created by Photoshop on a Windows computer. Presentation software (Neurobehavioral Systems) was used to present the stimuli and control the program. Stimuli were presented on a 19-inch Dell monitor with a screen resolution of 1,024 × 768 pixels and a refresh rate of 60 Hz. A chin and headrest were used to maintain the fixed viewing distance between the monitor and participants at approximately 57 cm. Participants used a keypad to respond.

#### Procedure

First, all participants were instructed to complete two tasks in the experiment: a training task and a cueing task. In the training task, the participants were instructed to associate two arrows, a red arrow and a green arrow, with “self” and “other,” respectively, and then they performed the cueing task using the self-and other-referential arrows. After two tasks, the participants were given instructions to complete a recognition task. The participants did not know that they needed to perform a recognition task until they completed the cueing task.

**Figure 1:**
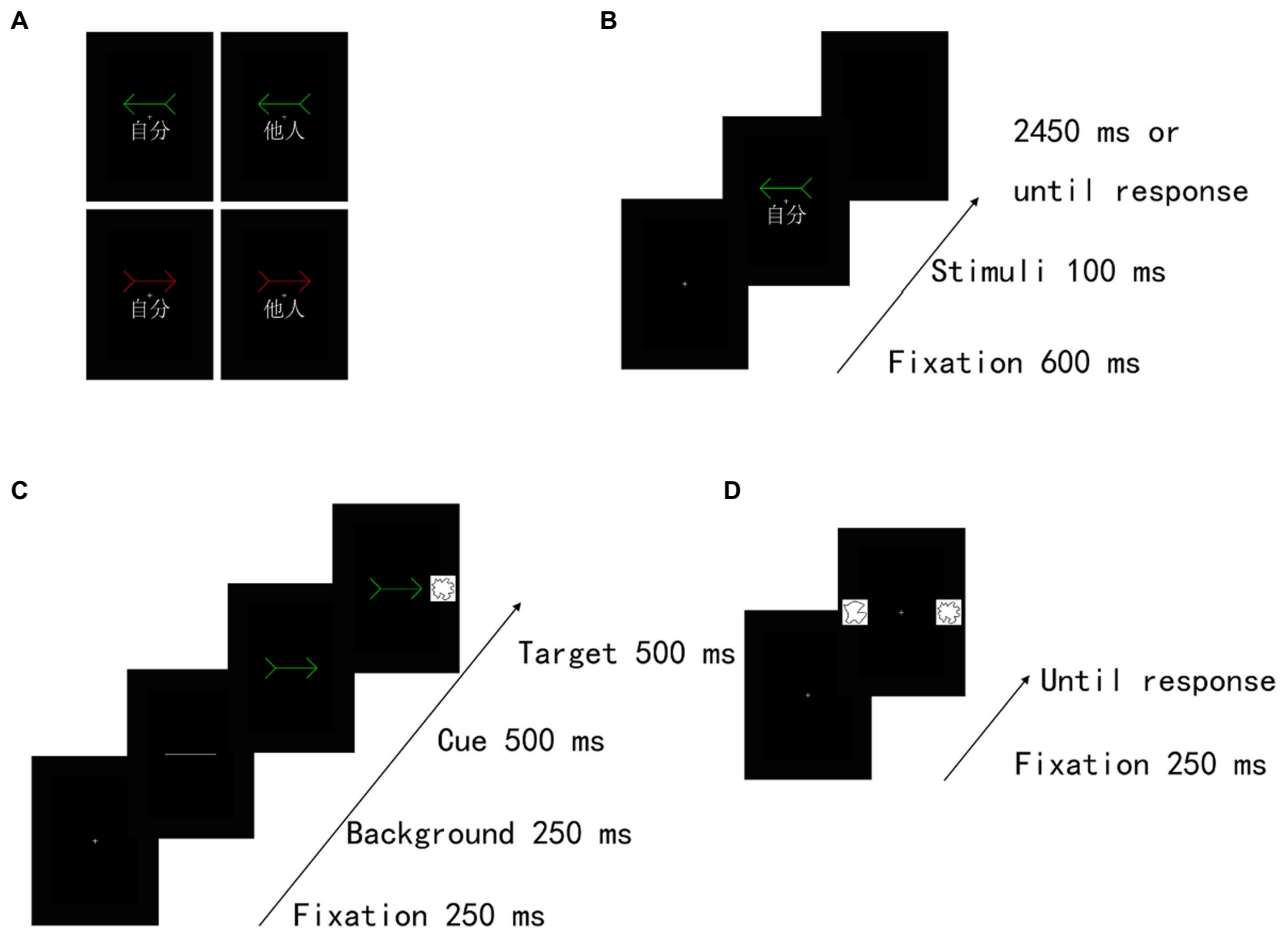
Experimental task structure in Experiment 1. **(A)** Examples of self-and other-arrow pair stimuli. Illustration of stimuli presented in the **(B)** training task, **(C)** cueing task and **(D)** recognition task. Two different colour arrows (i.e., red and green) were associated with the “self” or “other” words in the training task. Subsequently, a cueing task and a recognition task were implemented. In the cueing task, the participants were instructed to indicate as quickly and accurately as possible whether the target was presented on the left or right side by pressing the corresponding key (judge the location of the target). The target was equally likely to be presented on the same (valid cue condition) or opposite (invalid cue condition) side of the cue stimulus. The task consisted of 4 conditions, including valid and invalid conditions with self-and other-referential cues. In the recognition task, the participants were instructed to choose which of two shapes was displayed regardless of the location of the target in the cueing task. The recognition task consisted of 4 conditions, validity in the cueing task (valid and invalid cue condition) × self referentially in the cueing task (self-and other-reference conditions). The familiar shape was presented on the same (valid cue condition) or opposite (invalid cue condition) side as the self-or other-referential arrow cue in the cueing task.

Training task. The participants were instructed to associate self-and other-referential information (the word “self” [自分] or “other” [他人]) with different colour arrows (a red or a green arrow) (Figure 1A). As shown in Figure 1B, training trials began with a fixation cross at the centre of the screen presented for 600 ms. Then, the training stimulus (a red arrow or a green arrow) with an assigned or unassigned word was shown for 100 ms. We manipulated one of two different patterns between two colour arrows and the words (i.e., a red arrow associated with “self” and a green arrow associated with “other; a green arrow associated with “self” and a red arrow associated with “other”) for each participant. The participants were asked to judge whether the association between the arrow and the assigned word was correct. Specifically, although four stimulus pairs were included in total (a red arrow and self, a green arrow and other, a green arrow and self, a red arrow and other), we requested that the participants push a button only for the two correct stimulus pairs (e.g., a red arrow and self and a green arrow and other) as quickly and accurately as possible, but the other stimulus pairs (e.g., a green arrow and self and a red arrow and other) were requested to not push a button. Moreover, across participants, the assignments of these patterns (a red arrow and self, a green arrow and other, a green arrow and self, a red arrow and other) were counterbalanced. A block of 64 trials was performed with the self-related stimuli and other-related stimuli occurring equally often in a randomized order. Thirty-two practice trials preceded the experimental trials.

##### Cueing task

After the training task, a cueing task was immediately implemented. To ensure an effective orienting effect and interaction with memory, the procedure of the cueing task was based on the previous studies (Dodd et al., 2012). In the cueing task, the same arrow stimuli were used for the self-and other-referential cues; the stimulus presentation sequence in the cueing task is shown in Figure 1C. Each trial began with the appearance of a fixation cross for 250 ms at the centre of the screen, and then a transverse white line was presented for 250 ms at this location as a background. Subsequently, a cue stimulus pointing right or left (red or green arrow) was presented in the centre of the screen; the SOA between a cue event and a target event was 500 ms. A shape target stimulus was displayed to the left or the right spatial location of the cue for 500 ms. Two different sets of shape stimuli were used in the self-and other-referential cue conditions. The participants were instructed to indicate as quickly and accurately as possible whether the target was presented on the left or right side by pressing the corresponding key. Then, the reaction time (RT) to localize the target was recorded in each trial. The cue and the target remained present until response or until 2,500 ms had elapsed. The target was equally likely to be presented on the same (valid cue condition) or opposite (invalid cue condition) side of the cue stimulus. The central cues were uninformative and did not predict the target spatial location, and the participants were requested to keep their fixating screen centre. Similar to a previous study (Dodd et al., 2012), the task consisted of one block of 32 trials. Eight trials were performed under each condition. Each condition was presented in a pseudorandomized order.

##### Recognition task

Consistent with a previous study (Dodd et al., 2012), a memory task started immediately following the cueing task. Thus, after the cueing task, a recognition task started immediately with a short instruction. As shown in Figures 1D, a trial began with the appearance of a fixation cross for 250 ms at the centre of the screen. Then, two shapes were presented on either side of a black background. Specifically, one was drawn from two sets of shape stimuli presented in the cueing task, and the other was a novel stimulus drawn from another two sets. The participants were instructed to respond regarding which one of two shapes was displayed regardless of the location of the target in the cueing task. The task consisted of one block of 32 trials. Eight trials were included under each condition. Each trial was presented in a pseudorandom order.

#### Data analysis

For the training task, we measured total error rates (TERs), including omission and commission errors, to assess the strength of the association between arrows and self-or other-referential words using a cut-off of 10% error. Consistent with a previous study (Zhao et al., 2015), the participants were instructed to respond correctly on at least 58 trials in each block. RTs became stable after nearly 60 training trials, comparable to the results reported by Sui et al. (2009). Thus, we suggest that if the participants respond correctly on at least 58 trials in each block, then they have effectively learned the association between the “self” or “other” words and arrows stimuli. We excluded trials with abnormal RTs that were shorter than 150 ms or longer than 1,000 ms (0.9% of the trials). For each participant, the mean RTs and accuracy between self-and other-referential arrow conditions were analysed using a paired t test. The TERs of three (two males and one female) participants were greater than 10% and were excluded from the analysis. Thus, the association between arrow cues and “self” and “other” words was firmly established in the remaining participants.

In the cueing task, we excluded RTs that were shorter than 150 ms or longer than 1,000 ms (0.43% of the trials) and incorrect responses from RT analysis (0.06% of the trials). The error rates showed a floor effect because of a low rate of incorrect responses. Hence, we did not analyse the error data. The mean RT differences were analysed using a repeated-measures analysis of variance (ANOVA) with cue (self-and other-referential arrows) and validity (valid and invalid) as the within-participant factors. The resultant perceptual preferences of items were not different among cue type (self-referential cue or other-referential cue) and validity (valid or invalid) conditions under the cueing task in the remaining 51 participants (see Supplementary Table S2; Supplementary Results S2).

In the recognition task, the mean differences in accuracy rate for memorized items were analysed using a repeated-measures ANOVA with cue (self-and other-referential arrows) and validity (valid and invalid) as the within-participant factors. Then, to determine whether self-referential processing enhances or inhibits memory for items during attention orienting and memory performance under each condition, we examined whether the accuracy rate significantly differed from chance level (50%) using one-sample t tests.

### Results and discussion

#### Training task

The remaining 51 participants responded significantly faster to the arrow associated with the “self” word than to the arrow associated with the “other” word (mean ± SD, self: 523 ms ± 73.40 vs. other: 565 ms ± 77.28; CI: 95% confidence interval, self: 502.7–544.4 ms vs. other: 543.4–587.3 ms), *t* (50) = −6.15, *p* < 0.001, although the error rates were not significantly different between conditions (mean ± SD, self:0.21 ± 0.11% vs. other:0.43 ± 0.12%; CI: 95% confidence interval, self: 0–0.43% vs. other:0.18–0.68%), *t* (50) = −1.36, *p* = 0.18. Self-referential information has a stronger processing priority than other-referential information.

#### Cueing task

**Figure 2:**
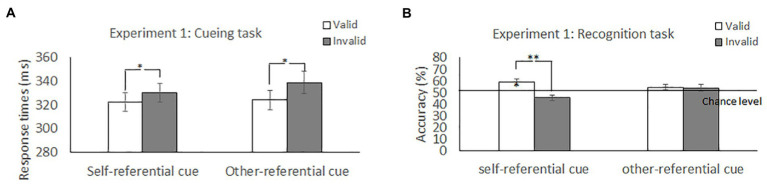
Mean difference in response times in the cueing task and accuracy in the recognition task to self-and other-referential arrow cues in Experiment 1. **p < 0.01; *p < 0.05.

Table 1 and Figure 2 show the mean RTs and error rates under each condition. We explored the validity effect using a 2 (cue type: self, other) × 2 (validity: valid, invalid) repeated-measures ANOVA. The analysis did not show a significant main effect of cue type, *F* (1, 50) = 2.04, *p* = 0.16, *η_p_^2^* = 0.04, or a significant cue type × validity interaction, *F* (1, 50) =0.83, *p* = 0.37, *η_p_^2^* = 0.02; however, we found a significant main effect of validity, *F* (1, 50) = 10.25, *p* = 0.02, *η_p_^2^* = 0.17, indicating that the RTs were faster in the valid condition than in the invalid condition.

**Table 1:** Mean response times (ms) in the cueing task and mean accuracy (%) in the recognition task as a function of cue and validity in Experiment 1.

Cue and validity	Cueing task				Recognition task		
	*M*	SEM	%E (SD)	CI	*M*	SEM	CI
Self-relevant arrow							
Valid	322.1	7.6	0.49 (2.4)	306.6–337.6	59.1	2.9	53.2–64.9
Invalid	329.7	7.8	0 (0)	313.8–345.6	45.6	2.5	40.5–50.6
Other-relevant arrow							
Valid	323.9	8.3	0.49 (2.4)	307.2–340.7	54.7	2.5	49.7–59.6
Invalid	338.6	9.5	0.49 (2.4)	319.3–357.8	53.9	2.9	48.0–59.8

M, mean; SEM, standard error of the mean; SD, standard deviation; %E, percent error rate; CI, 95% confidence interval.

#### Recognition task

The effect of self-referential processing was explored using a 2 (cue type: self and other) × 2 (validity: valid and invalid) repeated-measures ANOVA (Table 1 and Figure 2). The analysis revealed a significant main effect of validity, *F* (1, 50) = 10.78, *p* = 0.002, *η_p_^2^* = 0.18, indicating that accuracy for memory was higher in the valid condition than in the invalid condition; however, we did not find a significant main effect of cue type, *F* (1, 50) = 0.58, *p* = 0.45, *η_p_^2^* = 0.012. Notably, the cue type × validity interaction was significant, *F* (1, 50) = 5.19, *p* = 0.03, *η_p_^2^* = 0.09. *Post hoc* t tests found that the accuracy for memory was significantly greater for the valid condition than for the invalid condition for the self-referential arrow (*p* = 0.001) but not for the other-referential arrow (*p* = 0.81). The results indicated that the memory for the cued items was enhanced during attention orienting when using arrow cues associated with a “self” word.

Accuracy was also significantly higher than chance level for the valid condition when paired with a self-referential arrow cue [*t* (50) = 3.09, *p* = 0.003] but not when paired with other conditions [all *t* (50) < 1.88, *p* > 0.05]. The results indicated that memory performance was above chance level only when using arrow cues associated with a “self” word.

### Discussion

In the training task, the remaining participants almost always responded correctly (> 90%) and on at least 58 trials with arrows associated with words. The association was firmly established between arrow cues and (“self” and “other”) words. Additionally, participants responded faster to the arrows associated with “self” than to those associated with “other.” Consistent with previous studies (Zhao et al., 2018), a stronger processing priority was found for self-referential cues than for other-referential cues (arrows). In the cueing task, no significant difference in RTs to the target items was found between self-and other-referential cues. Participants reliably oriented their attention to the cued direction irrespective of self-and other-referential cues. In the recognition task, memorizing items presented at valid locations was higher than that presented at invalid locations when paired with a self-referential arrow cue but not when paired with an other-referential arrow cue. The memory for the cued items may be enhanced by self-referential processing even when participants did not attempt to memorize items following attention orienting. Given a potential difference between self and other words in the level of social salience, Experiment 2 investigated whether the social salience of the stimulus is self-relevant and can enhance memory during attention orienting.

## Experiment 2

The purpose of Experiment 2 was to determine whether the social salience of the stimulus is only self-relevant and can enhance memory during attention orienting.

### Experiment 2A

In Experiment 2A, participants were first asked to associate arrow cues with two different faces representing a happy or neutral emotion in the training task. To this end, a cueing paradigm was implemented, in which using the arrows associated with happy and neutral emotions as cues required the participants to determine whether various targets were presented at a right or left location. Finally, a recognition task was used to examine the effect of a cue associated with a happy face on memory even when participants did not attempt to memorize items during attention orienting. We hypothesized that the number of memorized items indicated by the cues could be enhanced when participants perceived arrow cues associated with happy faces (a high magnitude of social salience degree and a positive emotional valance) but not cues associated with neutral faces (a low magnitude of social salience degree and a neutral emotional valance).

#### Materials and methods

##### Participants

A different cohort of 54 naïve participants (mean age ± SD, 21.3 ± 1.52; 26 males) participated in Experiment 2A. All participants provided written informed consent before participating in the experiment, and all reported normal colour vision and normal or corrected-to-normal visual acuity.

##### Stimuli, apparatus, procedure, and analysis

**Figure 3:**
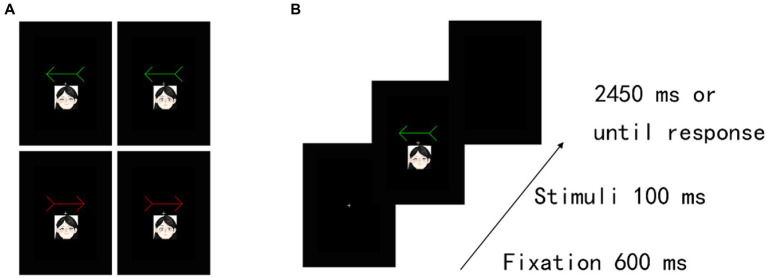
Experimental task structure and results in Experiment 2A. **(A)** Examples of happy-and neutral-arrow pair stimuli. **(B)** Illustration of stimuli presentation in the training task. Two different colour arrows (i.e., red and green) were included. These arrows were associated with a happy face or a neutral face in the training task. The actual stimuli were photographs of emotional faces from the KDEF database of faces (see Lundqvist et al., 1998).

The stimuli and procedure were identical to those in Experiment 1, except that we presented two emotional faces (happy and neutral) (3.8° wide × 4.6° high) with a straight gaze that was associated with a red arrow and a green arrow. The face stimuli (female, AF01; male, AM11) were taken from the Karolinska Directed Emotional Faces (KDEF) database of faces (Lundqvist et al., 1998). Both female and male emotional faces were displayed to all participants. Thus, the assignments of the patterns between two emotional faces, including female and male happy faces, female and male neutral faces, and red and green arrows, were counterbalanced across participants. Moreover, the emotional arousal of happy faces is 3.80 ± 1.85 SD, and that of neutral faces is 2.31 ± 1.47 SD (Goeleven et al., 2008). Thus, the level of social salience was different between happy and neutral faces. In the training task (Figure 3A), we excluded RTs that were shorter than 150 ms or longer than 1,000 ms (2.56% of the trials) from the analysis. The TER of one female participant’s data was greater than 10% in at least one block, and her data were excluded from the analysis. For the remaining participants, the results showed that the association was firmly established between arrow cues and emotion (happy and neutral faces).

Furthermore, in the cueing task (Figure 3B), we excluded RTs that were shorter than 150 ms or longer than 1,000 ms (0.12% of the trials) and incorrect responses (0.47% of the trials) from the analysis. Accuracy scores indicated a floor effect because of a low rate of incorrect responses. Hence, we did not analyse the error data. For the assigned target items under the cueing task, we confirmed no significant difference in perceptual preference among conditions under the cueing task in the remaining 53 participants (see Supplementary Table S3; Supplementary Results S3).

#### Results

##### Training task

The remaining 53 participants responded significantly faster to the arrow associated with a happy face than to the arrow associated with a neutral face (mean ± SD, happy face: 565 ms ± 99.37 vs. neutral face: 609 ms ± 77.05; CI: 95% confidence interval, happy face: 538.9–592.0 ms vs. neutral face: 587.5–630.4 ms), *t* (52) = −6.46, *p* < 0.001. Moreover, there was a significantly lower error rate in response to the arrow associated with a happy face than to the arrow associated with a neutral face (mean ± SD, happy:0.06 ± 0.04% vs. neutral:0.32 ± 0.10%; CI: 95% confidence interval, happy face: 0–0.14% vs. neutral face:0.12–0.52%), *t* (52) = −2.44, *p* = 0.019. A cue associated with a happy face had a stronger processing priority than a cue associated with a neutral face.

##### Cueing task

**Figure 4:**
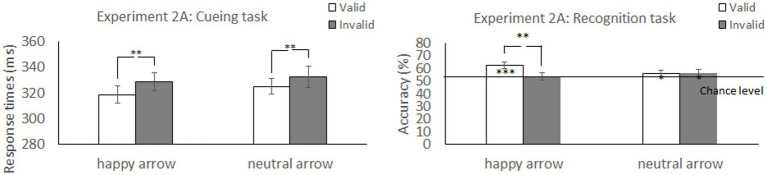
Mean difference in response times in the cueing task and accuracy in the recognition task to happy face-associated and neutral face-associated arrow cues. ***p < 0.001; **p < 0.01; *p < 0.05.

Table 2 and Figure 4 show the mean RTs and error rates for each condition. We explored the validity effect using a 2 (cue type: happy, neutral) × 2 (validity: valid, invalid) repeated-measures ANOVA. The analysis did not show a significant main effect of cue type, *F* (1, 52) = 2.37, *p* = 0.13, *η_p_^2^* = 0.04, or a significant cue type× validity interaction, *F* (1, 52) =0.10, *p* = 0.75, *η_p_^2^* = 0.002; however, there was a significant main effect of validity, *F* (1, 52) = 7.39, *p* = 0.009, *η_p_^2^* = 0.12, indicating that RTs were faster in valid conditions than in invalid conditions. Both arrows associated with a happy face and a neutral face reliably oriented attention in the cued direction.

**Table 2:** Mean response times (ms) in the cueing task and mean accuracy (%) in the recognition task as a function of cue and validity in Experiment 2A.

Cue and validity	Cueing task			Recognition task		
	*M* (SEM)	%E (SD)	CI	*M*	SEM	CI
Happy arrow						
Valid	318.5 (6.8)	0.94 (5.3)	304.8–332.2	62.5	2.5	57.4–67.6
Invalid	328.8 (7.0)	0.34 (1.7)	314.6–342.9	53.5	2.9	47.7–59.4
Neutral arrow						
Valid	324.9 (6.1)	0.47 (2.4)	312.5–337.2	55.9	2.5	50.8–61.0
Invalid	332.3 (8.2)	0.24 (0.23)	315.7–349.0	56.1	2.5	50.2–62.1

M, mean; SEM, standard error of the mean; SD, standard deviation; %E, percent error rate; CI, 95% confidence interval.

##### Recognition task

We explored the validity effect using a 2 (cue type: happy and neutral) × 2 (validity: valid and invalid) repeated-measures ANOVA (Table 2 and Figure 4). The analysis did not show a significant main effect of cue type, *F* (1, 52) =0.57, *p* = 0.46, *η_p_^2^* = 0.01, or validity, *F* (1, 52) = 3.45, *p* = 0.07, *η_p_^2^* = 0.06. Notably, there was a significant interaction between cue type and validity, *F* (1, 52) = 4.31, *p* = 0.04, *η_p_^2^* = 0.08. The accuracy for memory was significantly greater under the valid condition than under the invalid condition when a happy face was associated with a cue (*t* (52) = 2.77, *p* = 0.008) but not when a neutral face was associated with a cue (*t* (52) = −0.07, *p* = 0.94). The results indicated that the memory for the cued items was enhanced during attention orienting when using arrow cues associated with a happy face.

The accuracy for memorizing items was also significantly higher than chance level for the valid condition [*t* (52) = 4.96, *p* < 0.001] but not for the invalid condition [*t* (52) = 1.22, *p* = 0.23] when paired with the arrow associated with a happy face. When paired with the arrow associated with a neural face, accuracy was significantly higher than chance level for the valid [*t* (52) = 2.31, *p* = 0.025] and invalid conditions [*t* (52) = 2.07, *p* = 0.044]. The results indicated that memory performance was above chance level under both valid and invalid conditions when using arrow cues associated with a neutral face.

### Experiment 2B

Although Experiment 2A showed that the memory for the cued items was enhanced only when using arrow cues associated with a happy face but not a neutral face during attention orienting, the difference between happy and neutral faces exists not only in social salience but also in emotional valence. Thus, Experiment 2B examined whether the phenomenon was influenced only by positive emotional valence during attention orienting. Participants were trained to associate arrow cues (red and green arrows) and two different emotional stimuli representing a positive and a negative emotion (i.e., happy and fearful faces) prior to a cueing and a recognition task. We hypothesized that the number of memorized items indicated by the cues could be enhanced when participants perceived cues associated with happy (a high magnitude of social salience degree and a positive emotional valance) and fearful faces (a high magnitude of social salience degree and a negative emotional valance).

#### Materials methods

##### Participants

A different cohort of 54 naïve participants (mean age ± SD, 21.9 ± 3.3; 31 males) participated in Experiment 2B. All participants provided written informed consent before participating in the experiment, and all reported normal colour vision and normal or corrected-to-normal visual acuity.

##### Stimuli, apparatus, procedure, and analysis

**Figure 5:**
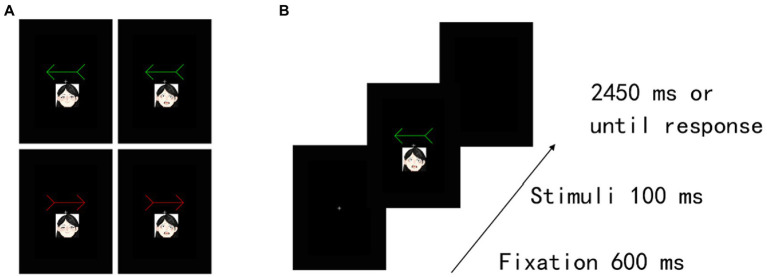
Experimental task structure and results in Experiment 2B. **(A)** Examples of happy-and fearful-arrow pair stimuli. **(B)** Illustration of stimuli presentation in the training task. The actual stimuli were photographs of emotional faces from the KDEF database of faces (see Lundqvist et al., 1998).

The stimuli and procedure were identical to those in Experiment 1, except that we presented two emotion faces (happy and fearful), each with a straight gaze, to be associated with a red and a green arrow. The Emotional arousal of score for the happy face was 3.80 ± 1.85 SD, and that of the fearful faces was 3.83 ± 1.66 SD (Goeleven et al., 2008). Thus, the level of social salience was similar for happy and neutral faces. In the training task (Figure 5A), we excluded RTs that were shorter than 150 ms or longer than 1,000 ms from the analysis (3.22% of the trials). The TERs of four (4 males) participants’ data were greater than 10% in at least one block, and their data were excluded from analysis. The results for the remaining participants showed that the association was firmly established between arrow cues and “self” and “other” words. Moreover, in the cueing task (Figure 5B), we excluded RTs that were shorter than 150 ms or longer than 1,000 ms (0.38% of the trials) and incorrect responses (0.44% of the trials) from the analysis. Accuracy scores existed for a floor effect because of a low rate of incorrect responses in the cueing task. Hence, we did not analyse the error data. For the assigned target items under the cueing task, we confirmed no significant difference in perceptual preference among conditions in the remaining 50 participants (see Supplementary Table S4; Supplementary Results S4).

#### Results

##### Training task

The remaining 50 participants responded significantly faster to an arrow associated with a happy face than to an arrow associated with a fearful face (mean ± SD, happy: 597 ms ± 99.99, fearful: 654 ms ± 81.72; CI: 95% confidence interval, happy face: 570.7–623.5 ms vs. fearful face: 630.2–677.2 ms), *t* (49) = −8.151, *p* < 0.001. Moreover, a significantly lower error rate was observed when responding to an arrow associated with a happy face than to an arrow associated with a fearful face (mean ± SD, happy face:0.28 ± 0.10% vs. fearful face:0.84 ± 0.14%; CI: 95% confidence interval, happy face:0.08–0.48% vs. fearful face:0.56–1.13%), *t* (49) = −3.397, *p* = 0.001. A cue associated with a happy face has a stronger processing priority than a cue associated with a fearful face.

##### Cueing task

Table 3 shows the mean RTs and error rates for each condition. We explored the validity effect using a 2 (cue type: happy and fearful) × 2 (validity: valid and invalid) repeated-measures ANOVA. The analysis did not show a significant main effect of cue type, *F* (1, 49) =0.299, *p* = 0.587, *η_p_^2^* = 0.006. Notably, there was a significant cue type × validity interaction, *F* (1, 49) = 4.23, *p* = 0.045, *η_p_^2^* = 0.08, and a main effect of validity, *F* (1, 49) =14.28, *p* < 0.001, *η_p_^2^* = 0.23, indicating that RTs were faster in valid conditions than in invalid conditions. The *post hoc* test revealed significantly shorter RTs for the valid condition than for the invalid condition for both an arrow cue associated with a happy face (*p* = 0.026) and an arrow cue associated with a fearful face (*p* < 0.001). There was no significant simple main effect of cue type under either valid (*p* = 0.37) or invalid conditions (*p* = 0.11). Both arrows associated with a happy and a fearful face reliably oriented attention to the cued direction, although the latter had a larger effect on attention orienting.

**Table 3:** Mean response times (ms) in the cueing task and mean accuracy (%) in the recognition task as a function of cue and validity in Experiment 2B.

	Cueing task			Recognition task		
Cue and Validity	*M* (SEM)	%E (SD)	CI	*M*	SEM	CI
Happy arrow						
Valid	311.3 (5.7)	0.5 (2.4)	299.7–322.9	54.8	2.4	50.2–59.8
Invalid	322.7 (6.9)	0 (0)	308.6–336.7	50.0	2.8	44.3–55.7
Fearful arrow						
Valid	307.1 (6.1)	0.5 (2.4)	294.8–319.4	55.3	2.5	50.2–60.3
Invalid	331.1 (6.4)	0.75 (3.9)	318.2–344.1	48.5	3.1	42.3–54.7

M, mean; SEM, standard error of the mean; SD, standard deviation; %E, percent error rate; CI, 95% confidence interval.

##### Recognition task

**Figure 6:**
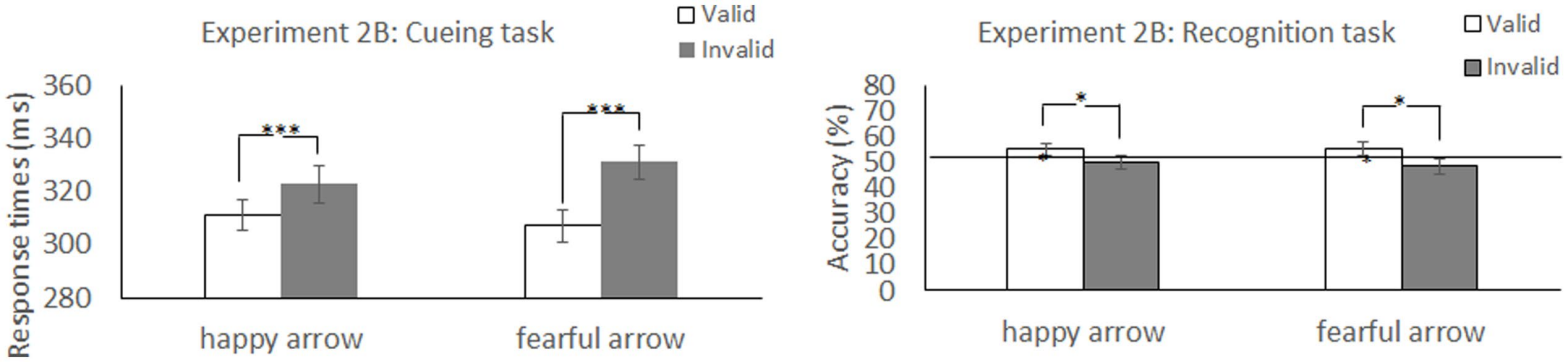
Mean difference in response times in the cueing task and accuracy in the recognition task to happy face-associated and fearful face-associated arrow cues. ***p < 0.001; *p < 0.05.

We explored the validity effect using a 2 (cue type: happy and fearful) × 2 (validity: valid and invalid) repeated-measures ANOVA (Table 3 and Figure 6). The analysis revealed a significant main effect of validity, *F* (1, 49) = 5.35, *p* = 0.025, *η_p_^2^* = 0.09, indicating that memory performance was higher in the valid condition than in the invalid condition; however, we did not show a significant main effect of cue type, *F* (1, 49) =0.06, *p* = 0.80, *η_p_^2^* = 0.001, or a significant cue type × validity interaction, *F* (1, 49) =0.11, *p* = 0.74, *η_p_^2^* = 0.002. The results indicated that memory for the cued items was enhanced during attention orienting when using arrow cues associated with a happy or a fearful face.

We found that accuracy was significantly higher than chance level for the valid condition [*t* (49) = 2.11, *p* = 0.04; *t* (49) = 2.10, *p* = 0.04] but not for the invalid condition [*t* (49) < 0.001, *p* = 1.0; *t* (49) = −0.49, *p* = 0.63] when using arrows associated with both happy and fearful faces as cues. The results indicated that memory performance was above chance level under valid conditions when using arrow cues associated with a happy face or a fearful face.

### Discussion

For the training task, the remaining participants almost always responded correctly (>90%) to arrows associated with emotional faces (“happy” and “neutral”) in Experiment 2A and (“happy” and “fearful”) in Experiment 2B. The association of arrow cues with face stimuli was firmly established, as shown in Experiment 1. A faster and more accurate response to the arrows associated with happy faces than to those associated with neutral faces in Experiment 2A and to those associated with fearful faces in Experiment 2B. Compared with neutral and negative facial expressions, participants were more sensitive to positive emotion. Consistent with this result, a previous study suggests a high level of asymmetry in the recognition and categorization among emotional signals (Leppänen and Hietanen, 2004). Specifically, compared with neutral and fearful faces, happy faces include low-level features, making them visually more salient (e.g., Hess et al., 1997) and resulting in an enhanced response to a happy face.

In the cueing task, no significant difference in the cueing effect was found between cue types in Experiment 2A (cues associated with a happy or a neutral face). Although a greater magnitude of cueing effect (i.e., invalid versus valid conditions) was shown when using arrows associated with fearful rather than happy faces as cues (*p* = 0.045) in Experiment 2B (Supplementary Results S5), the *post hoc* analysis for significant interaction did not reveal any clear difference between cue types under either valid or invalid conditions. This finding is consistent with a previous report that the cueing effect triggered by gaze direction is not influenced by static emotional faces, including neutral, happy and fearful faces (e.g., Hietanen and Leppänen, 2003), although an enhanced cueing effect by gaze direction was reported when using dynamic emotional faces compared with neutral faces (e.g., Uono et al., 2009; Lassalle and Itier, 2015). Moreover, a greater cueing effect was found for a fearful face than for a happy face when using a threatening stimulus as a target, whereas this greater cueing effect for fearful faces disappeared when using a pleasant stimulus as a target (Friesen et al., 2011; Kuhn and Tipples, 2011). Cueing effects could be influenced by emotional faces depending on participants’ goals. A set of meaningless shapes used as targets might obscure the difference in magnitude in the cueing effect between emotional faces.

Importantly, an interaction for accuracy was found in the recognition task. The results of Experiment 2A showed that memory performance for items was enhanced at valid locations but was not inhibited at invalid locations when the arrow associated with a happy face was used as a cue but not when a neutral face was used as a cue. Moreover, the results of Experiment 2B showed that memory performance for items presented at valid locations was enhanced but not inhibited at invalid locations when the arrows associated with happy or fearful faces were used as cues. When participants perceived stimuli associated with others’ happy or fearful faces with a straight gaze, either the positive or negative emotional valence of these emotional faces could enhance memory encoding during attention orienting. Thus, we suggest that during attention orienting, the memory for the cued items was enhanced only when using arrow cues associated with the magnitude of social salience regardless of emotional valence.

## General discussion

Following the firm establishment of the associations between specific stimuli (words and emotional faces) and arrow cues in the training task, the cueing task did not show a difference in the cueing effect between arrow cues associated with a high and a low magnitude of social salience stimulus, although both cues induced attention orienting (i.e., RTs were facilitated in valid conditions rather than in invalid conditions). Sui et al. (2009) investigated whether attention orienting was influenced by self-referential cues. The results showed more rapid attentional disengagement from the cued location to capture a target when using self-vs. friend-arrow cues with a short SOA. Zhao et al. (2015) implemented two types of targets (voice and tone) to examine the priority of self-referential processing during attention orienting. A facilitated cueing effect on the voice target relative to the tone target induced by self-referential but not other-referential cues. We proposed the possibility that the number of trials was not enough to detect the effect of self-referential processing and emotional processing in the present cueing task. To avoid a ceiling effect in memory, we used a small number of trials for each condition (i.e., 8 trials). Participants could see each target stimulus once in the cueing task. Previous studies including a large number of trials for each condition (e.g., 48 trials in Zhao et al., 2015) showed a significant behavioural difference in the cueing effect between self-and other-referential cues. Moreover, compared with these previous study settings (various SOAs and types of targets), a relatively simple design of the present cueing paradigm was used and may cause difficulties in distinguishing the difference in the cueing effect. However, this paradigm can effectively induce differences in memory performance during the subsequent recognition task.

Notably, an interaction for the accuracy was found in the recognition task. Given a different level of social salience for self-vs. other-referential processing (Sui et al., 2012; Scheller and Sui, 2022) and another person’s emotional face [A reviewer for Yiend (2010)], the recognition task suggested that the prioritization of social salience could facilitate memory following attention orienting. Previous studies (Bayliss and Tipper, 2006; Dodd et al., 2012) have shown an enhanced depth of encoding for valid targets, including incidental episodic memory and affective judgement, under gaze cues but not arrow cues. A qualitative difference exists between gaze and arrow cues. In contrast with arrows, the importance of other people’s eye gaze could modulate one’s own performance and influence one’s understanding of others’ intentions and interests. Based on these findings, in the present study, it can be interpreted that directional cues have been qualitatively modulated for the depth of encoding for valid targets by both social salience for self-referential words and emotional faces during attention orienting. We propose that the arrow stimuli associated with the high priority of social salience might trigger a qualitatively different behavioural performance for the depth of encoding for valid targets that is similar to that elicited by social cues (i.e., eye gaze) during the cueing task to memorize items due to the experiences that the participant had during the training task.

Given that this study examined social salience for self-referential words and emotional faces and that an interaction with attention orienting subsequently modulated the depth of memory encoding for the valid targets, our findings suggest that social salience was influenced by not only information associated with self-relevant stimuli (e.g., “self” words) but also the contained degree of salience in non self-relevant emotion stimuli (e.g., a happy face with a straight gaze); thus, social salience could also act as a modulator of processing information in social environments. Additionally, it would be useful to understand the importance of social salience as a potential mechanism underlying gaze-triggered attention orienting because a phenomenon similar to that observed with eye gaze was shown with self-referential and emotional arrow cues to facilitate the depth of memory encoding for valid targets during attention orienting. Additionally, given individuals can learn to associate reward with a colour, and subsequently prioritize this colour in the absence of said reward in a later task (e.g., Anderson et al., 2011), future research should examine whether not only social salience for self-referential words and emotional faces can also trigger an enhanced phenomenon (e.g., memory reward etc.)

The present findings might provide a clue for understanding how to prioritize the selection of relevant information in the environment, and then affect the depth of memory encoding. Some researchers (a review for Santangelo et al., 2015) have shown that memory can be modulated by the perceptual and semantic saliency of objects during the encoding of natural scenes. For example, the representation of short-term memory was enhanced by emotional information (Buttafuoco et al., 2018) and semantic congruence (Almadori et al., 2021). The present study showed that the depth of memory encoding for valid targets could be modulated by the social salience of the cue associated with self-referential words and emotional faces through attention orienting. This suggests that a cue stimulus (e.g., arrow and gaze) can orient one’s attention to an object in the environment and that intrinsic and/or experience-dependent semantic salience of the cue also influences the depth of memory encoding of the attended object. This extended mechanism might play an important role in learning about the social world under joint attention with (e.g., gaze and pointing gesture) or without other individuals (e.g., arrow and schematic gaze), resulting in individual differences in long-term memory representation.

The current findings also have implications for understanding impaired social attention in individuals with autism spectrum disorder (ASD). The impairment in social attention orienting (e.g., gaze-triggered orienting) has been characterized in individuals with ASD (e.g., Ristic et al., 2005; Goldberg et al., 2008; Marotta et al., 2013). However, most experimental evidence has reported generally intact gaze-triggered orienting in ASD (a review by Nation and Penny, 2008). Some researchers have recently highlighted the importance of a self-relevant component during attention orienting in individuals with typical development (TD) and ASD. For example, Zhao et al. (2018) showed that individuals with ASD exhibit intact self-referential processing but that self-referential processing affects the attention orienting of individuals with ASD in atypical patterns from that of TD individuals. Thus, one promising area of future research is the investigation of the impact of the social salience of cues for words and emotional faces on memory following attention orienting in individuals with ASD. The use of our paradigm may provide a possible design to explain atypical social attention orienting in individuals with ASD. For example, impaired social attention orienting might be influenced by atypical sensitivity to social salience in individuals with ASD.

The present study has some limitations that should be addressed. First, the accuracy is overall very close to chance level. Given a two-alternative forced choice task was implemented in recognition task, future research should use a Yes-No discrimination task and the *d’* prime analyse for measuring sensitivity and response bias, and also incorporate participants’ confidence ratings following each trial in the recognition task to evaluate their response confidence. Second, the participants’ biasing was influenced by the colours of the arrows. Although the RT of the social salience of word/emotion type was not influenced by the colour of the arrow in the cueing task across experiments, memory of the social salience of word/emotion type was influenced by the effects of the colour of the arrow in the recognition task in Experiments 1and 2B but not in Experiment 2A (Supplementary Results S6). Moreover, although the assignments of these colour patterns across participants were counterbalanced in all experiments, future research may need to investigate individuals’ preference scores for colours as a covariate to avoid participants’ biasing influenced by arrow colours. Finally, we did not use a neutral condition (e.g., no-cue condition) at baseline. A small number of trials were used for each condition (i.e., 8 trials) to avoid a ceiling effect in memory. Thus, future research may need to use a baseline condition in which the neural cues involve no spatial information, such as no-cue arrow, to determine how the depth of memory encoding for the valid target (i.e., facilitate or inhibit memory) was influenced by social salience of the cue associated with self-referential words and emotional faces.

Taken together, Experiments 1 and 2 provide the first evidence that the salience of social information works during attention orienting. Although a difference has been shown between self-referential processing and emotional processing in behaviour performance (e.g., no behavioural correlation between the self-referential bias and the emotional bias effects in Stolte et al., 2017), our results showed that the arrow stimuli associated with high social salience in Experiment 1 (self-referential information) and in Experiment 2A (emotional faces) might trigger a similar behavioural effect during a cueing task, facilitating the depth of memory encoding for valid targets due to the experiences of the participant during training. These findings suggest that a high degree of social salience for self-reference and emotional faces was found to facilitate the depth of memory encoding for the valid targets following attention orienting. In future research, we should investigate how self-referential processing and emotional processing are intertwined for social interaction (e.g., the depth of memory encoding under joint attention).

## Data availability statement

The datasets presented in this study can be found in online repositories. The names of the repository/repositories and accession number (s) can be found in the article/Supplementary material.

## Ethics statement

The studies involving human participants were reviewed and approved by the local ethics committee of Kyoto University Graduate School and Faculty of Medicine. The patients/participants provided their written informed consent to participate in this study.

## Author contributions

SZ: Conceptualization, Formal analysis, Writing Original Draft, Review & Editing; SU: Conceptualization, Writing Original Draft, Review & Editing; RH and SY: Conceptualization, MT: Conceptualization, Supervision. All authors contributed to the article and approved the submitted version.

## Funding

This study was supported by the Natural Science Foundation of Guangdong Province, China (Grant No. 2022A1515011167), Shenzhen Natural Science Fund (the Stable Support Plan Program 20200804111553001) and a Grant-in-Aid for Scientific Research (Japan Society for the Promotion of Science, grant 18K13368).

## Conflict of interest

The authors declare that the research was conducted in the absence of any commercial or financial relationships that could be construed as a potential conflict of interest.

## Publisher’s note

All claims expressed in this article are solely those of the authors and do not necessarily represent those of their affiliated organizations, or those of the publisher, the editors and the reviewers. Any product that may be evaluated in this article, or claim that may be made by its manufacturer, is not guaranteed or endorsed by the publisher.
