# Supplementary material for: Self-referential and social saliency information influences memory following attention orienting
Source: Front Psychol. 2023 Mar 22;14:1092512. doi: 10.3389/fpsyg.2023.1092512 (PMC10075135; doi:10.3389/fpsyg.2023.1092512)
Supplement: Supplementary file 1 [file Data_Sheet_1.pdf]

## Supplemental Data

### Supplemental Results

**Result S1**

Table S1 shows the mean perceptual preference scores of the four sets. The mean differences in perceptual preference scores for the 4 sets were analysed using a one-way ANOVA with conditions. Table S1 shows the mean perceptual preference scores under each condition. The analysis did not show a significant effect of sets, $F$ (3, 60) = 1.69, $p$ = .18.

**Result S2**

Table S2 Mean perceptual preference scores in the cueing task as a function of cue and validity in Experiment 1. We explored the validity effect using a 2 (cue type: self, other) × 2 (validity: valid, invalid) repeated-measures ANOVA with the remaining 51 participants. The analysis did not show a significant main effect of cue type, $F$ (1, 50) = .02, $p$ = .88, $\eta_p^2 < .001$, validity, $F$ (1, 50) = .16, $p$ = .69, $\eta_p^2 = .003$ or a significant cue type × validity interaction, $F$ (1, 50) = .01, $p$ = .91, $\eta_p^2 < .001$.

**Result S3**

Table S4 Mean perceptual preference scores in the cueing task as a function of cue and validity in Experiment 2A. We explored the validity effect using a 2 (cue type: self, other) × 2 (validity: valid, invalid) repeated-measures ANOVA with the remaining 53 participants. The analysis did not show a significant main effect of cue type, $F$ (1, 52) = .09, $p$ = .76, $\eta_p^2 = .002$, validity, $F$ (1, 52) = .017, $p$ = .896, $\eta_p^2 <.001$ or a significant cue type × validity interaction, $F$ (1, 52) = .002, $p$ = .97, $\eta_p^2 < .001$.

**Result S4**

Table S10 Mean perceptual preference scores in the cueing task as a function of cue and validity in Experiment 2B. We explored the validity effect using a 2 (cue type: self, other) × 2 (validity: valid, invalid) repeated-measures ANOVA with the remaining 50 participants. The analysis did not show a significant main effect of cue type, $F$ (1, 49) = .04, $p$ = .84, $\eta_p^2 = .001$, validity, $F$ (1, 49) = .05, $p$ = .83, $\eta_p^2 = .001$ or a significant cue type × validity interaction, $F$ (1, 49) = .001, $p$ = .97, $\eta_p^2 < .001$.

**Result S5**

To examine the cueing effect by differences between arrows associated with emotional

faces as cues in Experiments 2A and 2B, the cueing effect under condition (i.e., the RT improvement for the valid vs. invalid conditions) was analysed with a paired t test to compare emotional faces (happy vs. neutral face in Experiment 2A, happy vs. fearful face in Experiment 2B). The analysis did not show a significant difference in cueing effects of the happy and neutral face cues in Experiment 2A $t$ (52) = .317, $p$ = .752. However, a greater magnitude of cueing effect was found when using arrows associated with fearful rather than happy faces as cues in Experiment 2B $F$ (1, 49) = -2.056, $p$ = .045.

**Result S6**

We examined whether the social salience of word/emotion type was influenced by the effects of the colour of the arrow in both the cueing task and the recognition task in Experiments 1, 2A and 2B.

In Experiment 1, the mean differences were analysed using a repeated-measures analysis of variance (ANOVA) with cue (self- and other-referential arrows) and validity (valid and invalid) as the within-participant factors and colour pattern (red and green arrows) as the between-participant factors in both the cueing task and the recognition task. In the cueing task, ANOVA showed no significant interaction of word type × validity × colour pattern ($F$ (1, 49) = 2.908, $p$ = .094, $\eta_p^2$ = .056). In the recognition task, ANOVA showed a significant interaction of word type × validity × colour pattern ($F$ (1, 49) = 9.493, $p$ = .003, $\eta_p^2$ = .162). Post hoc t tests found that memory was significantly larger for the valid condition than for the invalid condition for the self-referential arrow ($p$ < .001) when using a green arrow as cue but not for the other conditions (all $p$ > .05)

In Experiment 2A, the mean differences were analysed using a repeated-measures ANOVA with cue (happy- and neutral-referential arrows) and validity (valid and invalid) as the within-participant factors and colour pattern (red and green arrows) as the between-participant factors in both the cueing task and the recognition task. In the cueing task, ANOVA showed no significant interaction of emotion type × validity × colour pattern ($F$ (1, 51) = .003, $p$ = .954, $\eta_p^2$ < .001). In the recognition task, ANOVA showed no significant interaction of emotion type × validity × colour pattern ($F$ (1, 51) = 1.77, $p$ = .19, $\eta_p^2$ = .033).

In Experiment 2B, the mean differences were analysed using a repeated-measures ANOVA with cue (happy- and fearful-referential arrows) and validity (valid and invalid) as the within-participant factors and colour pattern (red and green arrows) as the between-participant factors in both the cueing task and the recognition task. In the cueing task, ANOVA showed no significant interaction of emotion type × validity ×

colour pattern ($F$ (1, 48) = 3.324, $p$ = .075, $\eta_p^2$ = .065). In the recognition task, ANOVA showed a significant interaction of emotion type × validity × colour pattern ($F$ (1, 48) = 32.05, $p$ < .001, $\eta_p^2$ = .4). Post hoc t tests found that memory was significantly larger for the valid condition than for the invalid condition for the fearful-referential arrow ($p$ <.001) when using a red arrow as cue and for the happy-referential arrow ($p$ = .001) when using a green arrow as cue but not for the other conditions (all $p$ > .05).

**Supplemental Table**

**Table S1**

Mean perceptual preference scores in four sets.

| | M | SD |
|---|---|---|
| Condition | | |
| Set 1 | 4.62 | .35 |
| Set 2 | 4.61 | .30 |
| Set 3 | 4.40 | .27 |
| Set 4 | 4.56 | .29 |

M, mean; SD, standard deviation of the mean.

**Table S2**

Mean perceptual preference scores in the cueing task as a function of cue and validity in Experiment 1.

| | | Cueing task | |
|---|---|---|---|
| Cue and validity | | M | SD |
| Self-relevant arrow | Valid | 4.544 | .07 |
| | Invalid | 4.551 | .15 |
| Other-relevant arrow | Valid | 4.549 | .07 |
| | Invalid | 4.552 | .15 |

M, mean; SD, standard deviation of the mean.

**Table S3**

Mean perceptual preference scores in the cueing task as a function of cue and validity in Experiment 2A.

| | | Cueing task | |
|---|---|---|---|
| Cue and validity | | M | SD |
| Self-relevant arrow | Valid | 4.545 | .07 |

| | | | |
|---|---|---|---|
| | Invalid | 4.547 | .16 |
| Other-relevant arrow | Valid | 4.552 | .07 |
| | Invalid | 4.552 | .15 |

M, mean; SD, standard deviation of the mean.

**Table S4**

Mean perceptual preference scores in the cueing task as a function of cue and validity in Experiment 2B.

| | | Cueing task | |
|---|---|---|---|
| Cue and validity | | M | SD |
| Self-relevant arrow | Valid | 4.545 | .07 |
| | Invalid | 4.547 | .15 |
| Other-relevant arrow | Valid | 4.549 | .07 |
| | Invalid | 4.552 | .15 |

M, mean; SD, standard deviation of the mean.
